# Expanding the reach of HER2-targeted therapies: transformation of an historical paradigm

**DOI:** 10.1172/JCI166384

**Published:** 2022-12-15

**Authors:** Erin F. Cobain, Daniel F. Hayes

**Affiliations:** University of Michigan Rogel Cancer Center, Ann Arbor, Michigan, USA.

## Historical perspective on targeting HER2

Nearly four decades ago, King et al. reported that a novel candidate oncogene, *ERBB2* — also designated *c-neu* — which encodes human epidermal growth factor receptor 2 (HER2), is amplified up to 20-fold in a subset of human mammary carcinomas (1). HER2 is a member of the epidermal growth factor receptor (EGFR) family, which consists of three additional receptors — EGFR, HER3, and HER4. There are at least 11 ligands that bind to these three other receptors, but none has been identified for HER2 (2). Rather, HER2 signaling appears to be controlled by heterodimerization with the other EGFR tyrosine kinases.

Soon after this discovery, it was determined that *ERBB2* amplification and/or overexpression occurs in approximately 20% of all breast cancers and that amplification and overexpression corresponds with poor prognosis (3). This observation prompted substantial interest in the development of HER2-targeted therapeutics that might inhibit receptor dimerization, thereby inhibiting downstream cell growth and survival signals. In the early 1990s, a murine monoclonal antibody — initially designated mAb 4D5 — was developed against human HER2 and demonstrated promising effects in preclinical models, preventing HER2 dimerization and halting the growth of tumors overexpressing HER2 (4). mAb 4D5 was subsequently humanized and renamed trastuzumab. Shortly thereafter, a landmark clinical trial demonstrated a remarkable benefit to the combination of trastuzumab and chemotherapy versus chemotherapy alone in patients with metastatic breast cancer with HER2 overexpression (5). The success of this agent in the metastatic treatment setting led to multiple prospective randomized clinical trials of adjuvant trastuzumab in early-stage treatment of breast cancer, which demonstrated that chemotherapy plus trastuzumab reduced the risk of breast cancer recurrence by 9% and reduced breast cancer-specific mortality by 6.4% 10 years after diagnosis, when compared with chemotherapy alone (6).

## Expanding and improving HER2-targeted therapies

In the last 20 years, numerous other HER2-targeted therapies have proven successful in the metastatic, adjuvant and neoadjuvant settings, including another monoclonal antibody — pertuzumab — three separate tyrosine kinase inhibitors — lapatinib, neratinib, and tucatinib — and two antibody drug conjugates (ADCs) — ado-trastuzumab emtansine (T-DM1) and fam-trastuzumab deruxtecan (TDx-D). The ADCs represent a relatively new strategy in oncology, utilizing a site-specific monoclonal antibody to direct a highly potent, conjugated cytotoxic agent directly to cancer cells overexpressing the respective antigen (7). ADCs minimize systemic drug distribution and toxicity to normal cells, permitting improved drug delivery and optimization of the therapeutic index. Indeed, emtansine and deruxtecan have enormous toxicity as free drugs, making this mechanism of drug delivery paramount to safely administering these effective but toxic agents.

T-DM1 has been demonstrated to be a highly effective therapeutic strategy in first and later line settings in patients with metastatic HER2 positive breast cancer (8, 9). T-DM1 is also used routinely in the adjuvant treatment setting for those patients with early stage HER2 positive breast cancer who are at higher risk of disease recurrence based on the presence of residual disease at surgery following neoadjuvant systemic therapy (10). More recently, TDx-D was demonstrated to have substantial efficacy in patients with heavily pretreated metastatic HER2-amplified breast cancer, including those whose cancers had progressed on T-DM1 (11). Although a subgroup of patients (approximately 14%) experienced substantial pulmonary toxicity from this drug, the durable responses observed garnered strong enthusiasm for utilizing this therapy earlier in the treatment algorithm for this population. Indeed, results of a prospective randomized trial have established TDx-D as the preferred second line treatment for patients with metastatic HER2-overexpressed breast cancers (12).

## Who benefits from anti-HER2 therapies?

The remarkable success of trastuzumab and subsequently developed anti-HER2 therapies led to the development of a joint set of guidelines regarding HER2 analysis by the American Society of Clinical Oncology (ASCO) and the College of American Pathologists (CAP) (13). According to these guidelines, HER2 positivity is defined as either circumferential membrane staining that is complete, intense, and in greater than 10% of tumor cells (designated 3+ by IHC), or FISH with a HER2/CEP17 ratio of at least 2 and average HER2 copy number of at least 4 signals per cell. Using these criteria, CAP has established highly successful proficiency testing, which is required to achieve accreditation for clinical laboratories that perform HER2 testing (14).

Many investigators have wondered if the ASCO/CAP cutoffs for HER2 amplification and expression are clinically correct. Indeed, the development of TDx-D for patients with HER2–overexpressed metastatic breast cancer resurrected a previous theory that thresholds for defining HER2 positivity may need to be revisited. Indeed, following the publications of the adjuvant trastuzumab trials, Paik and colleagues in the National Surgical Adjuvant Breast and Bowel Project (NSABP) retrospectively analyzed HER2 expression levels on tumors from patients enrolled in the NSABP-B31 trial of adjuvant trastuzumab and identified 174 cases originally classified by their primary pathologists as being HER2 positive, but which lacked HER2 gene amplification upon central laboratory analysis (15). The relative benefits of adjuvant trastuzumab for these patients were similar to those with definitive HER2 amplification. Similar findings were also reported by Perez and colleagues from another of the adjuvant trastuzumab trials (16), and data from xenograft models indicated that trastuzumab may target the cancer stem cell population via a mechanism that does not require HER2 gene amplification (17).

These data prompted a subsequent prospective randomized trial of chemotherapy with or without trastuzumab (NSABP-B47) in patients with tumors for which HER2 IHC staining was 1–2+, but FISH failed to demonstrate amplification (18). Unfortunately, trastuzumab did not improve clinical outcomes for these patients, solidifying the paradigm that HER2-directed therapies should only be offered only to those patients with tumors with HER2 amplification or overexpression, as defined by ASCO/CAP guidelines.

Subsequent preclinical studies of TDx-D indicated that this agent might have activity in cancers that express HER2, but at much lower levels than required to be considered positive by ASCO/CAP guidelines. In these models, the bystander effect was observed; TDx-D was cytotoxic to cells neighboring those expressing HER2, due to the highly potent and membrane permeable payload of TDx-D (19). Therefore, it was determined that patients with HER2 1+ or 2+ disease might benefit from TDx-D, and phase II and subsequent phase III trials were initiated (11, 20). In the latter (the DESTINY-Breast04 study), patients with heavily pretreated HER2 1+ or 2+/FISH negative metastatic breast cancer were randomly assigned to TDx-D or physician’s choice of chemotherapy. Remarkably, progression-free survival was 9.9 months versus 5.1 months in the control group (hazard ratio for disease progression or death, 0.50; *P* < 0.001), and overall survival was 23.4 months versus 16.8 months for the control group (hazard ratio for death, 0.64; *P* = 0.001), favoring TDx-D (20). Based on these data, the FDA recently approved the use of TDx-D for patients with HER2 low–expression metastatic breast cancer who have progressed on at least one prior line of chemotherapy treatment, and these studies have changed our standard of care for this patient population.

## How much HER2 is needed for treatment efficacy?

The results of DESTINY-Breast04 have raised several important questions, as we consider the population of patients that may benefit from this therapy. The first question is whether TDx-D may have activity in all patients, regardless of HER2 expression level. This issue raises concern about pathologists’ ability to discern between HER2 IHC staining of 0 and 1+. It is possible that some cancers do not express HER2 at all (21). However, it seems more likely that the cancer expresses some HER2, but at an insufficient level to be detected by tissue staining methods. Furthermore, there may be very real differences in sensitivity and specificity among the antibodies at these very low levels of HER2, and, finally, different pathologists may read the stained tissue differently. A recent retrospective analysis of breast tumors in which 18 expert breast pathologists scored HER2 protein expression levels by IHC found that the concordance for scores between 0 and 1+ was quite poor, raising the concern that some patients may be missing out on the potential to benefit from TDx-D therapy (22). Importantly, the ASCO/CAP guidelines and the CAP proficiency testing were designed to designate overexpression compatible with trastuzumab activity, and not to distinguish low or normal expression from no expression of the HER2 protein. At present, this designation is critical to avoid both undertreating those patients whose cancers have some HER2 expression and not overtreating those who do not.

Given these concerns, it has been proposed that utilizing a quantitative assay measuring HER2 protein levels as a continuous variable would be a preferable strategy for selection of patients for treatment (23). Indeed, retrospective analyses using an automated reading of immunofluorescence staining indicate a broad range of HER2 protein expression, and up to two-thirds of breast tumors have had some evidence of expression, potentially broadening the population that could benefit from TDx-D (23, 24). While we currently recommend continuing to utilize IHC and FISH parameters in clinical practice for patient selection for treatment with TDx-D, we strongly advocate for incorporation of assays measuring HER2 protein expression levels more precisely into clinical trials, so that we can explore the possibility that novel assays may identify a broader population of patients able to benefit from this treatment.

It is of interest whether TDx-D might work even in patients with very low or even no HER2 expression. The results of the phase 2 DAISY trial, presented in abstract form only, have suggested that approximately one-third of patients whose tumors are determined to be HER2 0 by IHC had an objective response (21). A subsequent subanalysis of this trial in 10 patients has suggested that the responses may be related to substantial heterogeneity of HER2 expression that might result in misinterpretation of HER2 0 cases (25). Final analysis and publication of these results are pending, and at present we do not recommend the treatment of patients with TDx-D who have apparent HER2 IHC staining of 0.

## Balancing benefits with risks

Establishing which patients stand to benefit from treatment is critical in light of the toxicity of TDx-D. Pneumonitis occurs in 10–15% of patients, requiring cessation of therapy and administration of high dose steroids, and even sometimes resulting in death (26). Proactive monitoring and prompt initiation of treatment are recommended to decrease the severity of symptoms. This concern must be addressed as we move into the neoadjuvant treatment setting for both HER2 over- and underexpressed cancers, since many of these patients will likely be cured by standard therapies. Several trials are underway, including DESTINY-Breast05, DESTINY-Breast11 and the TALENT trial, all aiming to determine whether TDx-D may improve invasive disease-free survival among patients being treated with curative intent. Special attention will be given to the rates of pulmonary toxicity observed in these studies to weigh potential benefits and risks.

Finally, the potential mechanisms of resistance to TDx-D are poorly understood. While no definitive mechanisms of resistance have been uncovered to date, a number of mechanisms have been proposed (25), (a) decreasing HER2 expression levels; (b) alterations in the internalization of HER2, preventing effective delivery of the deruxtecan payload; (c) alterations to lysosomes, essential for the intracellular release of the deruxtecan payload; (d) increased expression of membrane-bound drug efflux pumps; and (e) intrinsic mechanisms of resistance to deruxtecan and other topoisomerase I inhibitors. Uncovering and simultaneously targeting these resistance mechanisms may further improve clinical outcomes.

## Conclusions

The understanding that HER2 plays a critical role in tumorigenesis has fundamentally changed the treatment of breast cancer over the last 30 years, with HER2-targeted therapies substantially improving the likelihood of survival and cure for a subset of patients. Introduction of ADCs, and particularly TDx-D, represents yet another major step forward in this field, but important questions remain concerning the selection of patients, management of toxicity, and mechanisms of resistance. Each of these is being addressed in ongoing preclinical studies, clinical trials, and reviews of current and potential policies and technologies in order to ensure that all patients who may benefit from this therapy are able to gain access to the treatment.

## Version 1 12/15/2022

Electronic publication
